# Peripheral *HMGB1* Downregulation Is Associated With the S100–IL‐17 Axis in Parkinson’s Disease: An Integrated Bulk and Single‐Cell Transcriptomic Analysis

**DOI:** 10.1155/padi/2128634

**Published:** 2026-09-30

**Authors:** Shuning Wang, Hang Shi, Xiangjun Meng, Hui Zhu

**Affiliations:** ^1^ Department of Neurology The First Hospital of Jilin University Changchun Jilin China jlu.edu.cn; ^2^ Department of Neurology Changchun Central Hospital Changchun Jilin China; ^3^ Department of Neurology Liaoyuan Central Hospital Liaoyuan Jilin China

**Keywords:** high-mobility group box 1, IL-17 signaling pathway, Parkinson’s disease, peripheral immunity, S100 gene family, single-cell RNA sequencing

## Abstract

**Background:**

Accumulating evidence implicates peripheral immune dysregulation in the pathogenesis of Parkinson’s disease (PD). High‐mobility group box 1 (HMGB1), a key alarmin released during mitochondrial stress, has been reported to be upregulated in the peripheral blood of PD patients, yet its precise role remains unclear.

**Methods:**

We performed an integrated analysis of multiple transcriptomic datasets from peripheral blood mononuclear cells (PBMCs) of PD patients. Using a combination of methods including machine learning, cell–cell communication analysis, in silico gene perturbation, and pathway enrichment analysis, we systematically investigated the expression and regulation of *HMGB1* in peripheral immune cells in PD.

**Results:**

We found that *HMGB1* mRNA expression was significantly downregulated in PBMCs from PD patients. A machine learning model identified *HMGB1* as a potential predictive signature (AUC = 0.685). Single‐cell transcriptomic analysis revealed immune cell heterogeneity and identified CD8^+^ T and CD4^+^ T cells as central hubs in the cellular communication network. In silico perturbation analysis suggested that *HMGB1* may be associated with the expression of *S100A8* and *S100A9*, which are enriched in the IL‐17 signaling pathway. Drug enrichment analysis identified potential therapeutic agents, including TPEN, hydrocotarnine, and phorbol 12‐myristate 13‐acetate.

**Conclusion:**

Our analysis revealed a distinct downregulation of *HMGB1* within the peripheral immune compartment of PD, a change that was decoupled from the overall proinflammatory state. Observing the concomitant upregulation of the S100 gene family and activation of the IL‐17 pathway, we propose a putative pathogenic axis based on computational findings: *HMGB1* downregulation is linked to S100 gene family upregulation and IL‐17 pathway activation.

## 1. Introduction

As a progressive neurodegenerative condition, Parkinson’s disease (PD) is pathologically defined by the irreversible degeneration of dopaminergic neurons within the substantia nigra. This neuronal loss underpins the development of a range of debilitating motor and nonmotor disabilities, which collectively impose a severe and growing burden on patients’ well‐being and healthcare infrastructures globally [1, 2]. Aberrant immune system activation has been recognized as a key link connecting multiple pathological processes. Neuroinflammation, particularly the chronic inflammatory response mediated by microglia, is widely acknowledged as a core factor accelerating neurodegeneration in PD [3, 4]. AS a key damage‐associated molecular pattern (DAMP), high‐mobility group box 1 (HMGB1) has emerged as a growing focus of research in PD [5–7]. Under homeostatic conditions, HMGB1 predominantly resides within the nuclear compartment, functioning in key genomic maintenance processes such as DNA damage repair and the modulation of gene transcription [5, 8, 9]. During cellular stress or damage, HMGB1 can be actively secreted or passively released, acting as an “alarmin” to activate pattern recognition receptors and initiate and amplify inflammatory cascades [8, 10]. Numerous studies have shown that extracellular HMGB1 can exacerbate microglial activation and neuroinflammation, leading to dopaminergic neuronal damage in PD animal models [10, 11], while inhibiting the HMGB1 signaling pathway exerts neuroprotective effects [12, 13]. These findings establish the classic proinflammatory role of HMGB1 within the central nervous system. However, the specific cellular and molecular mechanisms involved remain inadequately elucidated [14, 15]. In particular, it is unclear how different immune cell subsets and inflammatory mediators coordinate or act separately across the peripheral and central compartments. Therefore, a deeper understanding of the mechanisms underlying different immune cell subpopulations and the peripheral–central interplay in PD holds urgent clinical significance for the development of disease‐modifying therapies and early diagnostic tools.

To investigate the function of *HMGB1* in PD, we employed an integrated bioinformatics approach, analyzing transcriptomic data from peripheral blood mononuclear cells (PBMCs) of PD patients. A schematic of the complete analytical pipeline is provided in Figure 1. All computational work was implemented in the R statistical environment (Version 4.5.0).

**FIGURE 1:**
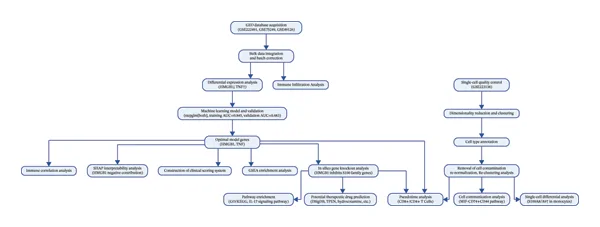
Study workflow. GEO, Gene Expression Omnibus; *HMGB1*, High Mobility Group Box 1; *TNF*, Tumor Necrosis Factor; AUC, Area Under the Curve; SHAP, SHapley Additive exPlanations; GSEA, Gene Set Enrichment Analysis; GO, Gene Ontology; KEGG, Kyoto Encyclopedia of Genes and Genomes.

## 2. Materials and Methods

### 2.1. Data Acquisition and Preprocessing

#### 2.1.1. Data Acquisition

We sourced the gene expression microarray data from the publicly accessible Gene Expression Omnibus (GEO) repository [16]. We selected three human PD PBMCs datasets, all generated using the Agilent‐014850 Whole Human Genome 4 × 44K microarray platform. Among them, GSE22491 (10 PD, 8 controls) and GSE75249 (14 PD, 13 controls) were combined as the training set, while GSE49126 (30 PD, 20 controls) served as the independent validation set. Additionally, a separate GSE223138 dataset (4 PD, 2 controls) was included for single‐cell RNA sequencing analysis independently.

##### 2.1.1.1. Ethical Statement

All analyses were performed using publicly available, de‐identified datasets from the GEO database. The original studies (GSE22491, GSE75249, GSE49126, and GSE223138) were approved by their respective institutional review boards, and informed consent was obtained from all participants. As such, additional ethical approval was not required for this secondary analysis.

#### 2.1.2. Batch Data Integration and Batch Effect Correction

Raw microarray data from GSE22491 and GSE75249 were quantile‐normalized and log2‐transformed. Probes targeting the same gene were collapsed using the avereps function (limma package, Version 3.66.0), and only genes present across all datasets were retained. Batch effect correction between the GSE22491 and GSE75249 datasets was then performed using the ComBat function (sva package, v3.58.0) [17], with batch labels defined by dataset of origin. Default parameters (including par.prior = TRUE) were used to remove technical variation while preserving biological signals. The efficacy of batch correction was subsequently evaluated through visual inspection of principal component analysis (PCA) plots, which were generated using the ggplot2 package (Version 4.0.2).

#### 2.1.3. Differential Expression Analysis

We first defined a gene set associated with mitochondrial membrane rupture and cell death. Differentially expressed genes (DEGs) within this gene set between PD and control groups in the training set (GSE22491 and GSE75249) were subsequently identified using the Wilcoxon rank‐sum test [18, 19]. *p* values derived from the analysis were adjusted for multiple comparisons via the Benjamini–Hochberg procedure, and genes meeting a false discovery rate (FDR) threshold of < 0.05 were defined as DEGs. We used the pheatmap package (v1.0.13) to generate a hierarchically clustered heatmap of the DEGs, and used the ggpubr package (v0.6.3) to plot expression distribution boxplots for key DEGs.

### 2.2. Single‐Cell RNA‐Seq Data Analysis

#### 2.2.1. Quality Control

The Seurat package (v5.4.0) was employed for the computational analysis of the single‐cell transcriptomic data [20]. During Seurat object creation, initial filtering was applied requiring that a gene be detected in at least 5 cells (min.cells = 5) and a cell express at least 100 genes (min.features = 100). The percentage of mitochondrial transcripts per cell (percent.mt) was then calculated using the PercentageFeatureSet function. For final quality control, cells were retained only if they satisfied nFeature_RNA > 100 and percent.mt < 10, thereby removing empty droplets and low‐quality cells [21].

#### 2.2.2. Normalization and Highly Variable Genes (HVGs) Selection

We performed log normalization on the total expression of each cell using the NormalizeData function with a scale factor of 10,000. Subsequently, we selected 1500 HVGs for downstream analysis using the FindVariableFeatures function [21].

#### 2.2.3. PCA and Batch Effect Correction

PCA was performed on the scaled expression matrix of the HVGs, retaining the top 20 principal components (RunPCA, npcs = 20). To integrate data across different batches, we applied batch effect correction using the harmony package (v1.2.0) [22].

#### 2.2.4. Dimensionality Reduction, Clustering, and Visualization

Based on the PCA‐reduced dimensions, we performed graph‐based clustering with the FindClusters function at a resolution of 0.6 to obtain biologically relevant clusters [23]. Finally, we generated a two‐dimensional visualization using t‐distributed stochastic neighbor embedding (t‐SNE) with the RunTSNE function.

#### 2.2.5. Removal of Cell Contamination, Renormalization, Dimensionality Reduction, and Clustering

Based on marker gene module scores (threshold > 0.2) [20, 24] and preliminary annotations, we removed contaminating nontarget cells (e.g., erythrocytes, platelets, and hematopoietic progenitors). Standard normalization, dimensionality reduction, and clustering procedures were then re‐executed on the remaining mature immune cells.

#### 2.2.6. Differential Expression Analysis and Cell Type Annotation

We used the FindAllMarkers function to identify cluster‐specific DEGs with the thresholds: FDR < 0.05 and |log2FC| > 1 [20]. Cell clusters were automatically annotated with the SingleR package (v2.12.0) [25] against seven locally precompiled human reference datasets. Annotation results were visualized using the scRNAtoolVis package (v0.1.0).

#### 2.2.7. Cell‐Cell Communication Analysis

We inferred intercellular ligand‐receptor interaction networks using the CellChat package (v1.6.1) [26]. Communication probabilities and their statistical significance for specific ligand‐receptor pairs across cell types were visualized using bubble plots.

### 2.3. Machine Learning Model Construction

Given the characteristics of our data and the limited sample size of the training set (*n* = 45), 15 machine learning algorithms—including support vector machine, Elastic Net, and regularized logistic regression variants—were preliminarily compared via 10‐fold cross‐validation using the caret package (v7.0.1) in R for preliminary screening, with the area under the receiver operating characteristic curve (AUC) as the primary metric. To mitigate potential optimism bias from algorithm selection and performance estimation on this small cohort, the optimal algorithm identified from this preliminary comparison was subsequently evaluated using nested 10‐fold cross‐validation on the training set (GSE22491 + GSE75249). Specifically, the outer folds provided an unbiased performance estimate, while all preprocessing and feature selection steps were strictly confined within the inner folds to prevent information leakage. The final model was refitted on the full training set and externally validated on an independent cohort (GSE49126). ROC curves and AUC values were generated using the pROC package (v1.19.0.1) to quantify classifier accuracy.

### 2.4. Interpretability Analysis of the Logistic Regression Model and Clinical Score Construction

To interpret the optimal model, we calculated SHapley Additive exPlanations (SHAP) values for each feature using the kernelshap package to quantify their contribution to predictions. Additionally, to evaluate clinical predictive performance, we constructed a nomogram and plotted the calibration curve using the rms package (v1.8.1).

### 2.5. Functional Enrichment and Immune Infiltration Analysis

To functionally interpret the expression signature of the optimal feature gene set, we applied Gene Set Enrichment Analysis (GSEA) via the clusterProfiler package (v4.18.4) [27], thereby interrogating its association with annotated biological pathways. Concurrently, we employed the CIBERSORT deconvolution algorithm to estimate the relative proportions of 22 immune cell types in each sample. Algorithm robustness was ensured via 1000 permutation tests. Furthermore, sample filtering was applied based on a CIBERSORT‐derived *p* value threshold (< 0.05) to select samples for subsequent analysis.

### 2.6. Cell Type‐Specific Expression Analysis

To visualize the expression patterns and cell‐type specificity of the optimal feature gene set in the single‐cell atlas, we used the Seurat package (v5.4.0).

### 2.7. In Silico Gene Perturbation and Pathway Analysis

To simulate the functional perturbation of the key gene and infer its downstream regulatory network, we performed in silico gene perturbation analysis on disease‐group single‐cell transcriptomic data (encompassing CD4+ T cells, CD8+ T cells, monocytes, B cells, NK cells, and dendritic cells) using the scTenifoldKnk algorithm [28]. The network was constructed using a pooled expression matrix from all disease‐group cells, without stratification by cell type or donor. This method virtually disrupts the key gene’s regulatory relationships, compares gene expression networks before and after perturbation, and identifies significantly affected genes. The predicted downstream genes served as input for Gene Ontology (GO) [29, 30] and Kyoto Encyclopedia of Genes and Genomes (KEGG) [31] pathway enrichment analyses using the clusterProfiler package [27], with an FDR < 0.05 defined as significant enrichment.

### 2.8. Pseudotime Trajectory Analysis

To explore the dynamic differentiation trajectories of specific T‐cell subsets (e.g., CD8^+^ T, CD4^+^ T) in PD and the expression changes of key genes during differentiation, we performed pseudotime analysis on the selected cells using the Monocle package (v2.30.0) [32].

### 2.9. Potential Therapeutic Drug Prediction

To identify small‐molecule compounds potentially capable of reversing the disease‐associated gene expression profile in PD PBMCs, we performed drug enrichment analysis on the DEGs from the in silico gene perturbation using the clusterProfiler package against a local DSigDB database [33]. This screened for compounds showing a significant negative correlation with the expression profile. The resultant drug‐gene interaction network was visualized using Cytoscape software (v3.10.0) [34].

## 3. Results

### 3.1. Data Integration and Differential Expression Identification

To correct for technical batch effects, we merged the GSE22491 and GSE75249 datasets using the ComBat algorithm. PCA revealed that after correction, samples clustered by disease status (PD vs. control) rather than by dataset origin, confirming successful batch effect removal (Figure 2A–D). Using this integrated dataset, we performed differential expression analysis (FDR < 0.05) on a predefined gene set associated with mitochondrial oxidative membrane rupture and death. This analysis identified significant downregulation of *ACTR2*, *ACTR3*, *HMGB1*, and *RICTOR*, coupled with upregulation of *TNF*, in PD patient PBMCs compared to healthy controls (Figure 3A). A hierarchically clustered heatmap further confirmed the robust and distinct expression patterns of these genes between the two groups (Figure 3B).

**FIGURE 2:**
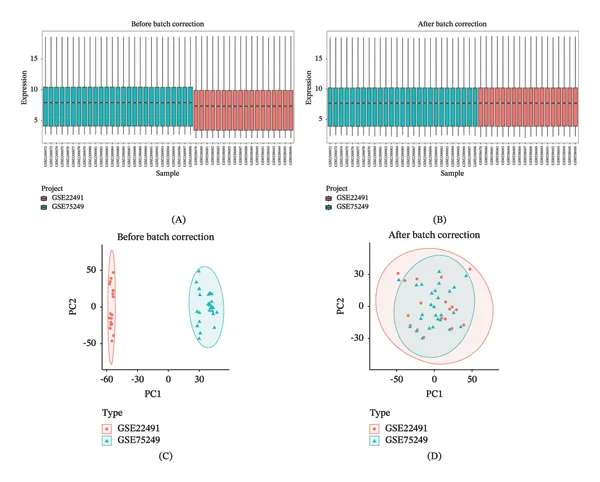
Transcriptomic data analysis before and after batch effect correction. (A) Expression distribution of samples before batch correction. (B) Expression distribution of samples after batch correction. (C) Principal component analysis (PCA) plot before batch correction, showing clear separation of samples from different datasets (GSE22491 and GSE75249). (D) PCA plot after batch correction, showing good mixing of samples from different datasets, indicating effective removal of batch effects.

**FIGURE 3:**
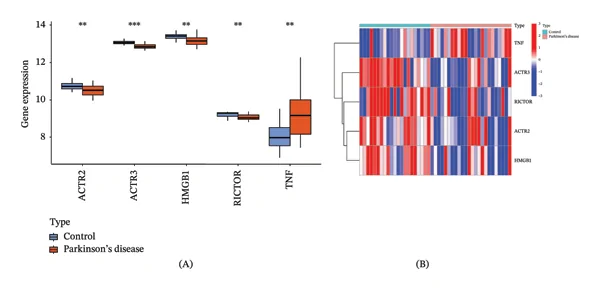
Expression differences and clustering analysis of target genes in Parkinson’s disease (PD) versus controls. (A) Boxplots showing expression levels of *ACTR2*, *ACTR3*, *HMGB1*, *RICTOR*, and *TNF* between the PD and control groups. ^∗∗^ *p* < 0.01;^∗∗∗^ *p* < 0.001. (B) Heatmap and hierarchical clustering based on the expression profiles of the above genes. Red indicates high expression, blue indicates low expression.

### 3.2. Single‐Cell Atlas and Immune Cell Communication

We utilized the publicly available single‐cell RNA sequencing dataset GSE223138, which comprises PBMC profiles from PD patients and matched healthy controls. Subsequently, rigorous preprocessing steps, including quality control and batch effect adjustment, were implemented to establish a reliable analytical foundation (Supporting Figure S1A). PCA confirmed the effective removal of batch effects (Supporting Figure S1B). Examination of the top genes associated with principal components (PCs) revealed that PC1 was associated with immune and metabolic functions, while PC2 was linked to immune responses (Supporting Figure S1C). Permutation tests confirmed that the observed intergroup differences were statistically significant (*p* < 0.05), ruling out the possibility that the observed variation was attributable to random chance or systematic error (Supporting Figure S1D). We selected 1500 HVGs for subsequent analysis (Supporting Figure S1E). T‐SNE dimensionality reduction demonstrated that cells clustered primarily based on their transcriptomic profiles rather than by disease status (Supporting Figure S2). Following the removal of potential cellular contaminants, a secondary validation of data quality and clustering integrity was performed. A second permutation test again supported the significance of the biological variation (Supporting Figure S1F), and a scree plot indicated that the first few principal components captured the majority of significant biological variance in the dataset (Supporting Figure S1G). After stringent quality control and validation, the processed single‐cell RNA sequencing dataset was deemed reliable for downstream investigation, including unsupervised clustering, cell type annotation, and functional exploration. Unsupervised clustering of PBMCs successfully resolved the major immune cell compartments (Figure 4A). To ascertain the biological identity and coherence of these clusters, we visualized the expression of the top 10 discriminatory marker genes per cluster in a heatmap (Figure 4B). Distinct lineage‐specific signatures emerged: CD8^+^ T cells were defined by *GZMH*, *CD8A*, and *CD8B*; CD4^+^ T cells by *CCR7* and *IL7R*; monocytes by *CD14*, *S100A8*, and *S100A9*; B cells by *PAX5*, *IGHD*, and CD24; and dendritic cells by *LAMP5* and *SCT*. Crucially, the expression profile of these genes corresponded precisely with the established canonical markers for each immune lineage, thereby validating the accuracy of our clustering and annotation strategy. Cell–cell communication analysis revealed a complex immune interaction network centered on CD8^+^ T and CD4^+^ T cells (Figure 4C–H), with the MIF signaling axis (via CD74 and CD44 receptors) emerging as a key mediator of communication both within T‐cell subsets and between T cells and other cell types (Figure 4I). Further, differential expression analysis across all annotated cell types was performed. Notably, monocytes were the only cell type exhibiting a significant and widespread upregulation of proinflammatory DAMP genes, including *S100A8*, *S100A9*, and *VCAN*. In contrast, CD4^+^ T cells, B cells, and dendritic cells showed a coordinated downregulation of these proinflammatory DAMP genes (Figure 4J).

**FIGURE 4:**
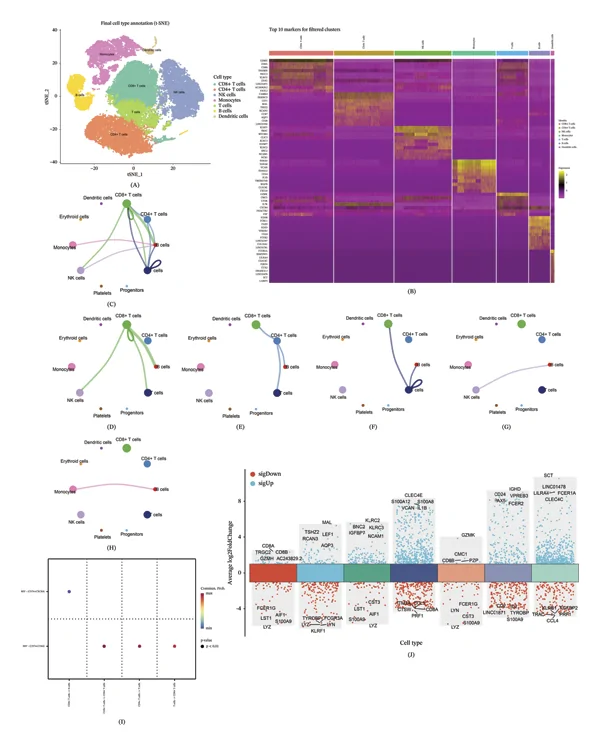
Cell type annotation and differential expression analysis of single‐cell sequencing data. (A) t‐distributed Stochastic Neighbor Embedding (t‐SNE) plot showing the final annotated cell types. (B) Heatmap showing expression of the top 10 marker genes for each cluster. (C) Integrated cell–cell communication network summarizing ligand–receptor interactions among cell types shown in panels (D–H). (D–H) Cell–cell communication network analysis between different cell subsets, with connecting lines representing ligand–receptor interactions. (I) Bubble plot of the cell–cell communication network, highlighting the MIF–(CD74+CD44) signaling pathway as a key driver of communication within T‐cell subsets. (J) Heatmap of marker gene expression across cell types. Red indicates downregulated expression, blue indicates upregulated expression.

### 3.3. Machine Learning–Based Biomarker Screening and Validation

Fifteen machine learning algorithms were preliminarily compared via 10‐fold cross‐validation on the training set (*n* = 45). The Stepwise Generalized Linear Model with bidirectional selection (Stepglm[both]) achieved the highest training AUC (0.845; Figure 5A) and was selected for further evaluation. Nested 10‐fold cross‐validation of Stepglm[both] yielded an AUC of 0.710 (Figure 5B, red dashed line). When applied to the independent validation cohort (GSE49126), the model achieved an AUC of 0.685 (Figure 5B, blue solid line). This model identified *HMGB1* and *TNF* as the two core feature genes distinguishing PD patients from controls. To interpret the model, we employed SHAP analysis. Results showed that the mean absolute SHAP value for *HMGB1* (0.162) was slightly higher than that for *TNF* (0.133), indicating a greater overall contribution of *HMGB1* to the model’s predictions. Specifically, higher expression of *HMGB1* was associated with a lower predicted risk of PD, whereas higher expression of *TNF* was associated with a higher predicted risk (Figure 5C). Finally, a nomogram was constructed based on the expression levels of *HMGB1 and TNF* (Figure 5D)*.* The calibration curve for the nomogram demonstrated high predictive accuracy, as the predicted probabilities closely tracked the actual observed PD risk along the ideal 45‐degree reference line (Figure 5E).

**FIGURE 5:**
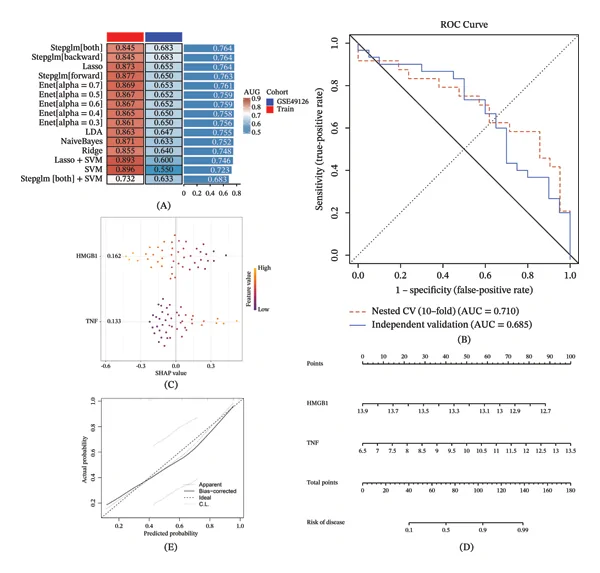
Construction and validation of the disease risk prediction model. (A) Comparison of AUC performance for different machine learning algorithms. (B) Receiver operating characteristic (ROC) curves of the Stepglm[both] for diagnosing PD. Red dashed line: Nested CV (10‐fold) (AUC = 0.710). Blue solid line: Independent Validation (AUC = 0.685). (C) Bee swarm plot of feature SHAP values. (D) Nomogram based on the biomarkers. (E) Calibration curve of the model.

### 3.4. Functional and Immune Microenvironment Characterization

GSEA showed that genes differentially expressed in the low *HMGB1* expression group were primarily enriched in metabolism‐ and neurodevelopment‐related pathways (Figure 6A). In contrast, genes in the high *TNF* expression group were markedly enriched in classic proinflammatory cascades, specifically the mitogen‐activated protein kinase (MAPK) and toll‐like receptor (TLR) signaling pathways (Figure 6B). Further assessment of immune cell infiltration uncovered significant remodeling within the PD immune microenvironment (Figure 6C,D). In patients with PD, the proportions of activated NK cells and activated mast cells were significantly increased, whereas the proportions of M0 macrophages and resting mast cells were significantly decreased (Figure 6C). Correlation analysis further revealed a strong positive association between *TNF* levels and multiple activated immune cell populations. Conversely, *HMGB1* expression correlated primarily with specific adaptive and innate immune subsets (Figure 6E). Cell type‐specific expression analysis showed that *HMGB1* was primarily highly expressed in adaptive immune cells and some innate immune cells, while *TNF* expression levels were very low in these subsets, suggesting differences in their regulatory mechanisms and functional roles (Figure 6F, G).

**FIGURE 6:**
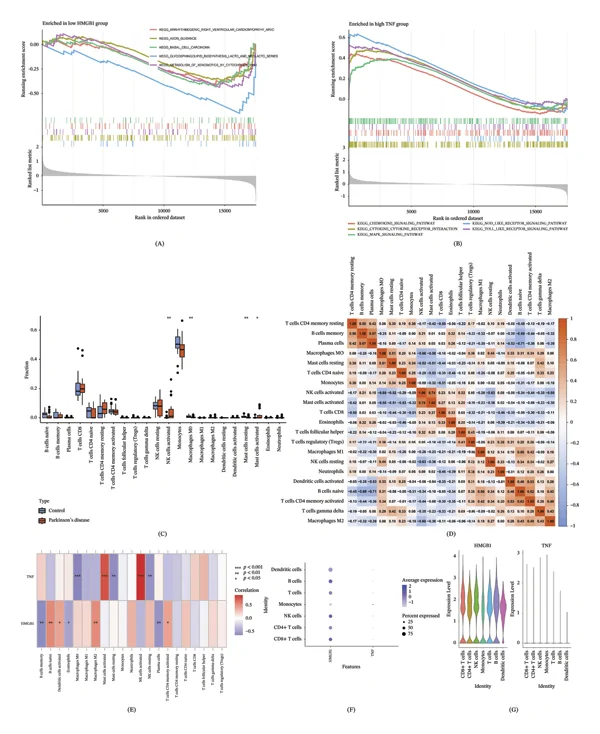
Functional and immune microenvironment analysis of *HMGB1* and *TNF* in PD. (A) GSEA enrichment plot for KEGG pathways in the low *HMGB1* expression group. (B) GSEA enrichment plot for KEGG pathways in the high *TNF* expression group. (C) Boxplots comparing differences in immune cell infiltration proportions between control and PD groups. (D) Heatmap of correlations among immune cells. Red represents positive correlation; blue represents negative correlation. (E) Correlation heatmap for *HMGB1*, *TNF*, and immune cells. (F) Scatter plots showing significant correlations between *HMGB1*/*TNF* and specific immune cells. (G) Violin plots showing expression levels of *HMGB1* and *TNF* across different cell types.

### 3.5. Regulatory Role of *HMGB1* and Cellular Trajectory Dynamics

To explore the potential downstream regulatory effects of *HMGB1*, we performed in silico gene perturbation analysis using the scTenifoldKnk algorithm. Results showed that simulated perturbation of *HMGB1* was associated with significant expression changes (FDR < 0.05) in only a few genes: members of the S100 gene family (*S100A8*, *S100A9*, *S100A12*) and *VCAN*, all of which were predicted to show significant upregulation (Figure 7A–C). Subsequently, we performed pseudotime analysis to delineate the differentiation trajectories of CD8^+^ and CD4^+^ T cells (Figure 8A–F). During this dynamic differentiation process, the expression of *S100A8* and *S100A9* was observed to be upregulated in the late differentiation stages, whereas the expression of *HMGB1* exhibited a downregulated trend (Figure 8A–F). *TNF* expression remained at relatively low levels throughout the differentiation trajectory (Figure 8B,E).

**FIGURE 7:**
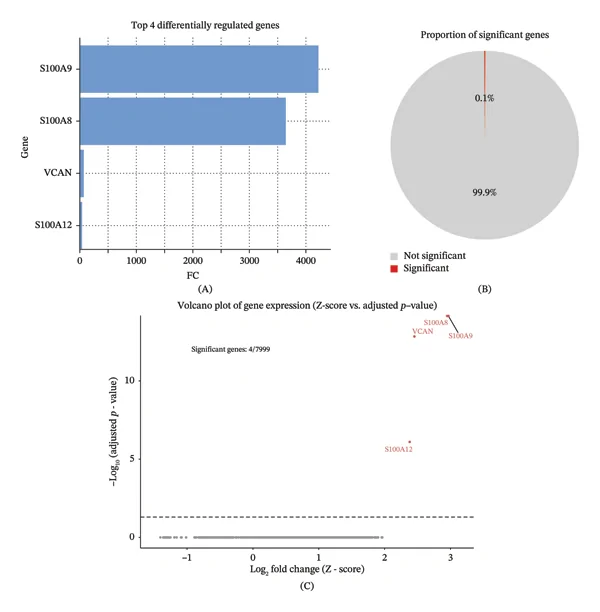
Results of the in silico gene perturbation simulation. (A) Bar chart of the top 4 differentially regulated genes ranked by fold change. (B) Pie chart showing the proportion of significantly differentially regulated genes (0.1% of genes met the significance threshold). (C) Volcano plot of differentially regulated genes. Red dots represent the 4 significantly differentially regulated genes (*S100A8*, *VCAN*, *S100A9*, and *S100A12*) identified from a total of 7999 analyzed genes.

**FIGURE 8:**
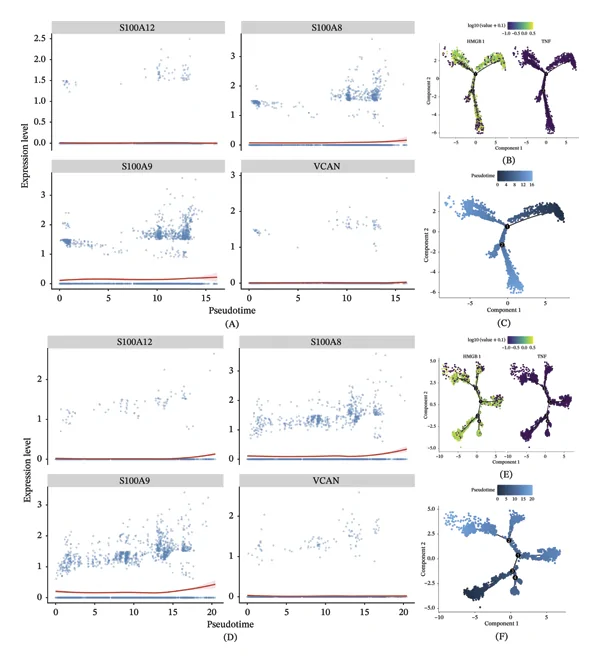
Expression dynamics of S100 family genes and pseudotime trajectory in CD8^+^ and CD4^+^ T cells. (A) Expression trends of *S100A12*, *S100A8*, *S100A9*, and *VCAN* genes along pseudotime in CD8^+^ T cells. (B) Distribution of *HMGB1* and *TNF* gene expression along the CD8^+^ T‐cell differentiation trajectory. (C) CD8^+^ T‐cell trajectory plot, colored by pseudotime. (D) Expression trends of the aforementioned genes along pseudotime in CD4^+^ T cells. (E) Distribution of *HMGB1* and *TNF* gene expression along the CD4^+^ T‐cell differentiation trajectory. (F) CD4^+^ T‐cell trajectory plot, colored by pseudotime.

### 3.6. Pathway Enrichment and Drug Prediction

To characterize the function of the differentially expressed genes (*S100A8*, *S100A9*, *S100A12*, and *VCAN*) identified via in silico gene perturbation, we conducted functional enrichment analysis. GO analysis showed these genes were significantly enriched in biological processes such as immune response and inflammatory response (Figure 9A). KEGG pathway analysis revealed significant enrichment in the IL‐17 signaling pathway and cornified envelope formation pathway (Figure 9B). We also performed drug enrichment analysis, which identified several small molecule compounds showing a significantly negative correlation with this gene expression signature, such as TPEN, Hydrocotarnine, and Phorbol 12‐myristate 13‐acetate (PMA), known to target *S100A8*, *S100A9*, *S100A12*, and *VCAN* (Figure 9C,D).

**FIGURE 9:**
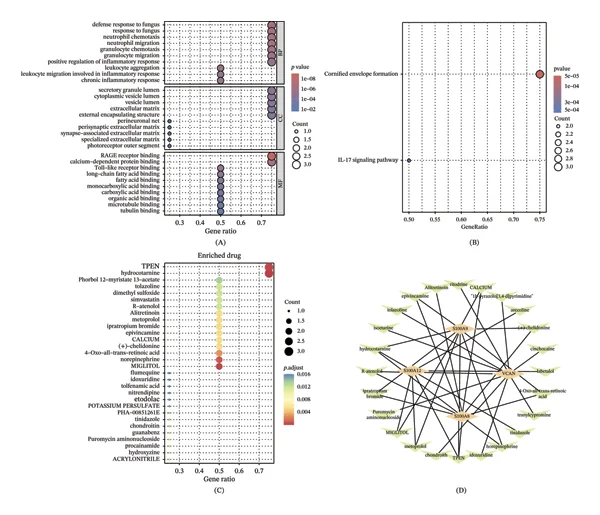
Functional enrichment analysis and drug interaction network. (A) GO enrichment bubble plot displaying the top 10 significantly enriched biological processes, cellular components, and molecular functions. (B) KEGG signaling pathway enrichment bubble plot. (C) Bar chart of enriched drugs and their adjusted *p* values. The compounds, identified via connectivity analysis, exhibit potential regulatory effects. (D) Interaction network diagram of key targets (*S100A8*, *S100A9*, *S100A12*, and *VCAN* gene) and enriched drugs.

## 4. Discussion

The pathogenesis of PD involves complex systemic and central immune dysregulation, but the connection between peripheral immune abnormalities and central neuroinflammation remains unclear [35, 36]. Although clinical studies indicate significantly elevated serum HMGB1 protein levels in PD patients, suggesting its involvement in systemic inflammation [11, 37]. The transcriptional regulatory pattern and functional significance of *HMGB1* within peripheral immune cells have not been fully elucidated [6]. HMGB1 is a critical output signal of mitochondrial stress [38]. Meanwhile, studies have shown that mitochondrial dysfunction is one of the key pathogenic mechanisms in PD [39]. Therefore, we propose the following hypothesis: peripheral immune cells may respond to mitochondrial stress and contribute to local and systemic inflammatory states through pathways potentially involving H*MGB1*‐mediated signaling. This “mitochondrial stress–*HMGB1*–immune dysregulation” axis may represent a putative link between key pathological events in PD, thereby offering a coherent framework connecting peripheral immune abnormalities to central nervous system pathology.

### 4.1. Downregulation of *HMGB1* in Peripheral Immunity and Potential Functional Decoupling

A central and unexpected finding of our study is the significant downregulation of *HMGB1* mRNA in the PBMCs of PD patients. This stands in stark contrast to the well‐documented elevation of HMGB1 protein in the serum of PD patients, as reported in prior studies [11, 37]. The coexistence of this transcriptional downregulation with the upregulation of *TNF* points to a distinct “expression–function decoupling” of HMGB1, challenging its canonical role as a uniformly proinflammatory alarmin [5, 8]. This further suggests that HMGB1 may be subject to intricate posttranscriptional regulation and functional reprogramming within the chronic inflammatory landscape of PD. Mechanistic insights into this decoupling emerged from our analyses. GSEA positioned samples with high *TNF* expression within robust inflammatory pathways (e.g., MAPK, TLR). Informed by this and established principles of inflammatory negative feedback [40], we propose a compensatory model wherein the sustained proinflammatory milieu may suppress *HMGB1* transcription. Concurrently, SHAP value analysis revealed that higher *HMGB1* mRNA levels correlated with a lower risk of PD, hinting at a protective function for its transcriptional activity. Consequently, the observed downregulation in patient PBMCs may signify a deleterious loss of this protective transcriptional program within the peripheral immune compartment. An alternative mechanistic explanation for the observed transcript‐protein decoupling may lie in the established principles of *HMGB1* posttranscriptional and translational regulation, as documented in prior studies [41]. This systemic decoupling may reflect an adaptive molecular mechanism that functionally diversifies immune cell populations, thereby potentially reconciling the differential activities observed at single‐cell resolution. Cell‐type‐specific differential expression analysis revealed a striking dichotomy in transcriptional responses to PD pathology: monocytes were specifically activated, with widespread upregulation of proinflammatory DAMP genes (e.g., *S100A8*, *S100A9*), potentially serving as inflammatory “amplifiers.” In contrast, cell types such as CD4+ T cells and B cells generally downregulated these genes, suggesting a potential “suppressor” role.

Final cell–cell communication network analysis indicated that CD8^+^ T and CD4^+^ T cells acted as central hubs, with particularly active intercellular interactions mediated via key pathways such as MIF–(CD74+CD44). In silico gene perturbation analysis further suggested that *HMGB1* may be associated with upregulation of the S100 gene family, potentially indicating an upstream regulatory relationship. Pseudotime analysis dynamically demonstrated that during the late differentiation stages of CD8^+^ and CD4^+^ T cells, downregulation of *HMGB1* expression coincided with upregulation of *S100A8* and *S100A9* expression. Functional enrichment analysis of differentially regulated genes (*S100A8*, *S100A9*, *S100A12*, and *VCAN*) identified from the in silico perturbation analysis was performed. GO analysis revealed that these genes were significantly enriched in immune defense response and inflammatory response pathways. KEGG pathway analysis further indicated their significant enrichment in the IL‐17 signaling pathway and the cornified envelope formation pathway.

Based on the static and dynamic analyses presented above, we propose the following hypothesis: upstream pathological signals in PD, such as mitochondrial stress, may induce the translocation and release of HMGB1 protein [42]. Sustained HMGB1 protein release and inflammatory signaling may, through a negative feedback loop, compensatorily suppress its own transcription, leading to decreased *HMGB1* mRNA levels in PBMCs and thereby establishing a state of “expression–function decoupling”. Within this context, different immune cell subpopulations may exhibit distinct responses to this signaling environment, characterized by high extracellular HMGB1 protein levels and low intrinsic *HMGB1* transcript levels. Specifically, in monocytes and terminally differentiated effector T cells, reduced *HMGB1* may be associated with derepression of DAMP molecules such as *S100A8* and *S100A9*, potentially amplifying local inflammation via the IL‐17 pathway. Conversely, in subpopulations such as CD4^+^ T cells and B cells, the overall downregulation of *HMGB1* coincides with a broad downregulation of proinflammatory genes, which may represent a compensatory transcriptional reprogramming by the immune system to limit inflammatory spread. In summary, this “expression–function decoupling” of HMGB1 may represent a key immunoregulatory switch in the peripheral immunity of PD patients, converting upstream stress signals into a downstream, highly heterogeneous and dysregulated immune response, and may represent an important mechanism underlying PD‐related systemic immune dysregulation.

### 4.2. *HMGB1* Forms an Axis Connecting Mitochondrial Stress to a Specific Inflammatory Network via Putative Negative Regulation of *S100A8* and *S100A9*

The most critical mechanistic clue in this study comes from the in silico gene perturbation analysis. We found that simulating *HMGB1* perturbation was associated with upregulation of S100 family DAMP genes, such as *S100A8* and *S100A9*. This supports the hypothesis that, in the peripheral immune cells of PD patients, *HMGB1* may act as an upstream negative regulator of *S100A8* and *S100A9*. Previous studies have shown that S100A8, S100A9, and HMGB1 all belong to the DAMP family and can synergistically amplify inflammatory signaling through receptors such as RAGE. This finding is further corroborated by other analyses in our study. Cell–cell communication analysis revealed a remodeled immune interaction network centered on CD8^+^ T cells and CD4^+^ T cells. Pseudotime trajectory analysis demonstrated that during the late differentiation stages of both CD8^+^ T and CD4^+^ T cells, the downregulation of *HMGB1* expression was accompanied by a corresponding upregulation of *S100A8* and *S100A9*. Pathway enrichment analysis revealed marked enrichment of the differentially regulated gene set within the IL‐17 signaling axis, identifying it as a core dysregulated pathway. By integrating the findings described above, we propose a “mitochondrial stress–*HMGB1*–S100–IL‐17 axis” hypothesis. Previous studies have shown that HMGB1 is a key responder to oxidative stress [43]. Therefore, we hypothesize that in the specific immune microenvironment of PD, mitochondrial stress may trigger the release or functional alteration of HMGB1. This could lead to the loss of HMGB1’s putative transcriptional repressive activity in certain immune cells, such as late‐stage differentiated effector T cells, thereby relieving its inhibition of DAMP genes including *S100A8* and *S100A9*. The upregulated *S100A8* and *S100A9* may then, through their receptors (e.g., RAGE), further promote the expression of proinflammatory pathways such as IL‐17 signaling [44–46]. It is also noteworthy that cell type‐specific differential expression analysis revealed widespread upregulation of proinflammatory DAMP genes such as *S100A8* and *S100A9* in monocytes, while these genes were generally downregulated in CD4^+^ T cells, B cells, and other subsets. We therefore hypothesize that different immune cell subpopulations may exhibit heterogeneous responses to the mitochondrial stress–*HMGB1* signaling axis. In monocytes and specific differentiation stages of T cells, reduced *HMGB1* signaling may be associated with transcriptional programs that upregulate proinflammatory genes such as *S100A8* and *S100A9*, potentially via receptors like RAGE, thereby contributing to proinflammatory responses. In other subsets such as CD4^+^ T cells and B cells, this signal may be directed toward distinct downstream pathways, ultimately linking to the observed downregulation of proinflammatory genes and their potential immunoregulatory functions. In summary, our study suggests a revised perspective on the role of *HMGB1* in the PD immune microenvironment: from a relatively nonspecific “alarmin” to a molecule that may finely tune the inflammatory state of distinct immune cell subpopulations through its putative regulatory effects on genes such as *S100A8* and *S100A9*.

### 4.3. Significance of the *HMGB1*/*TNF* Axis in Immune Microenvironment Remodeling, Cell Differentiation, and Therapeutic Prediction

Within the peripheral immune landscape of PD, *HMGB1* and *TNF* emerged as potential peripheral immune biomarkers. Despite this shared prominence, their biological roles diverge sharply. A high *TNF* expression signature correlated with an elevated presence of activated NK cells and mast cells, and its associated transcriptional program was robustly enriched in canonical inflammatory cascades such as MAPK and TLR signaling. This pattern reinforces the classic proinflammatory paradigm of TNF, wherein it primarily drives immune cell recruitment and activation. In contrast, *HMGB1* expression exhibited a positive association with regulatory cell subsets, including M2 macrophages. This association suggests a functional profile that extends beyond simple proinflammatory signaling, adopting a more “context‐dependent” role capable of dynamically modulating immune activity in response to varying oxidative states or microenvironmental cues. This functional complexity is further manifested in the cell differentiation process. Pseudotime analysis of CD8^+^ T‐cell and CD4^+^ T‐cell differentiation trajectories revealed a progressive downregulation of *HMGB1* expression, accompanied by a low‐level yet discernible upregulation trend in the canonical myeloid genes *S100A8* and *S100A9*. This dynamic shift may mark a critical juncture where T cells are either undergoing or are poised to undergo functional reprogramming within the PD microenvironment. Based on the inflammatory network associated with the “HMGB1‐low/S100‐high” gene signature, we further predicted potential compounds capable of reversing this gene signature via drug enrichment analysis, including TPEN, Hydrocotarnine, and PMA. We found that the mechanisms of action of these compounds align closely with the “mitochondrial stress–*HMGB1*–S100–IL‐17” axis proposed in our study. S100 amplifies proinflammatory signaling pathways by binding to its receptor RAGE [47]. Studies have shown that certain S100 proteins can bind zinc ions [48]. Therefore, we speculate that if zinc binding is structurally required for the formation of the S100–RAGE complex, zinc chelators such as TPEN may disrupt S100‐mediated amplification of proinflammatory signaling by sequestering zinc ions, thereby directly inhibiting complex assembly. Noscapine has demonstrated neuroprotective and anti‐inflammatory activity in PD models [49, 50]. Given that hydrocotarnine is a primary metabolite of noscapine, we speculate that part of noscapine’s beneficial effects may be mediated by hydrocotarnine. This speculation will need to be tested experimentally in future work. Studies have shown that PMA is a potent activator of protein kinase C, which may reverse gene expression profiles by altering the activation or differentiation state of T cells [51, 52]. We therefore speculate that PMA may reverse or reprogram the dysfunctional state of T cells in the peripheral blood of PD patients through the aforementioned mechanism, thereby exerting a therapeutic effect. Together, these findings suggest that TPEN, hydrocotarnine, and PMA may reverse disease‐characteristic gene expression profiles by targeting the S100 gene family and components of the immune microenvironment, offering a potential direction for the “drug repurposing” strategy aimed at PD‐associated immune dysregulation.

### 4.4. Study Strengths, Limitations, and Future Directions

#### 4.4.1. Strengths of Our Study

Our study is the first to systematically integrate multiple microarray gene expression profiles and single‐cell transcriptomic data from PD patient PBMCs. Using a multiangle computational validation strategy that combines machine learning, in silico gene perturbation, and pseudotime analysis, we comprehensively dissected the peripheral immune microenvironment in PD. Furthermore, we conducted an in‐depth exploration of the regulatory function of *HMGB1* and proposed a novel hypothesis linking the downregulation of *HMGB1* to the upregulation of the S100 gene family, suggesting that *HMGB1* may act as a negative regulator of S100 genes (e.g., *S100A8*, *S100A9*). This establishes a new axis connecting mitochondrial stress to inflammation.

#### 4.4.2. Limitations of Our Study

While this study provides new insights, its limitations must be considered. First, our analyses rely on public datasets (GSE75249 and GSE22491) in which individual‐level demographic and clinical metadata—including age, sex, disease duration, disease severity, medication status, and detailed sample‐processing procedures—were not available. Consequently, we were unable to adjust for these potential covariates in our analyses. These factors may influence PBMC composition and gene expression; therefore, the observed transcriptional differences between PD patients and controls could be partially attributable to demographic, clinical, or technical confounding across datasets. Second, the limited sample size of current PD PBMC transcriptomic studies constrains the generalizability of our findings, which is reflected in the moderate predictive performance of our machine learning model (AUC = 0.685 for the independent validation set; 0.710 in nested cross‐validation). Third, a methodological limitation of our in silico perturbation analysis is that the gene co‐expression networks were constructed using pooled cells from multiple immune lineages (e.g., T cells, monocytes). While this approach captures global regulatory trends within the peripheral blood immune system, the predicted regulatory relationship between *HMGB1* and S100 genes may be influenced by variations in cell‐type composition across samples and might be driven by specific subpopulations (e.g., monocytes) or indirect effects. More fundamentally, the key mechanistic hypotheses generated—such as the proposed *HMGB1*–S100 regulatory relationship and the “expression–function decoupling”—remain to be experimentally verified at the protein and functional levels. Finally, our findings are confined to the peripheral immune system. A critical unanswered question is how these peripheral alterations influence, or are influenced by, central neuroinflammatory processes. Addressing this will require integrating cerebrospinal fluid analyses or developing appropriate animal models in future work.

#### 4.4.3. Future Directions

The hypotheses and limitations arising from this work define a clear roadmap for future investigation. A priority is to validate the diagnostic utility of *HMGB1* and *TNF* in large‐scale, prospective PD cohorts with comprehensive clinical annotation—including age, sex, disease duration, disease severity, medication status, and sample‐processing details—to enable adjustment for potential confounding covariates. To spatially resolve the proposed pathogenic axis within relevant tissue architectures, advanced techniques such as spatial transcriptomics could be deployed. At the mechanistic level, functional studies are imperative to test two core premises: whether HMGB1 transcriptionally regulates the S100 gene family, and whether mitochondrial stress acts as the upstream trigger. Furthermore, the therapeutic potential suggested by our in silico predictions warrants experimental evaluation; the efficacy and mode of action of candidate compounds like TPEN, hydrocotarnine, and PMA should be assessed in preclinical PD models.

## 5. Summary

In summary, by integrating multiple bioinformatics approaches, this study is the first to identify the downregulation of *HMGB1* transcription and its decoupling from the overall proinflammatory state in the peripheral immunity of PD, and proposes a novel neuroinflammatory regulatory axis defined as “*HMGB1* downregulation ⟶ putative derepression of S100 gene family ⟶ IL‐17 pathway activation.” This finding provides a new perspective for understanding how mitochondrial stress may drive systemic inflammation through HMGB1 dysregulation in PD. However, this regulatory relationship remains to be experimentally validated. Future studies should focus on verifying this neuroinflammatory axis in experimental models and exploring its potential as a disease‐modifying target for PD therapy.

## Author Contributions

Conceptualization, Hui Zhu and Shuning Wang; methodology, Shuning Wang; software, Shuning Wang; validation, Shuning Wang, Hui Zhu, and Hang Shi; formal analysis, Shuning Wang; investigation, Shuning Wang; resources, Hui Zhu; data curation, Shuning Wang; writing–original draft preparation, Shuning Wang; writing–review and editing, Shuning Wang, Hui Zhu, Hang Shi and Xiangjun Meng; visualization, Shuning Wang, Hang Shi and Xiangjun Meng; supervision, Hui Zhu; project administration, Hui Zhu.

## Funding

No funding was received for conducting this study.

## Disclosure

All authors have read and agreed to the published version of the manuscript.

## Conflicts of Interest

The authors declare no conflicts of interest.

## Supporting Information

Additional supporting information can be found online in the Supporting Information section.

## Supporting information


**Supporting Information** Supporting Figure S1. Quality control and variance analysis of single‐cell RNA sequencing data. (A) Number of genes, transcripts, and mitochondrial gene percentage for each sample. (B) Principal component analysis (PCA) plot (PC1‐PC4). (C) Association of highly variable genes (HVG) with principal components (PC1–PC4). (D) Permutation test for PCA significance. (E) Selection of HVG. (F) Secondary permutation test for PCA. (G) Scree plot of principal component standard deviations. Supporting Figure S2. T‐distributed stochastic neighbor embedding (t‐SNE) dimensionality reduction and cell‐type annotation for single‐cell RNA sequencing data. Supporting Code S1. R script for batch correction and normalization pipeline. Supporting Code S2. R script for differential expression analysis pipeline.

## Data Availability

This study is a secondary bioinformatic analysis of publicly available data. The transcriptomic datasets analyzed in the current study, including GSE22491, GSE75249, GSE49126, and GSE223138, are publicly available in the NCBI Gene Expression Omnibus (GEO) repository. The custom R scripts used for data processing and analysis are available from the corresponding author upon reasonable request.
