# Supplementary material for: Peripheral HMGB1 Downregulation Is Associated With the S100–IL‐17 Axis in Parkinson’s Disease: An Integrated Bulk and Single‐Cell Transcriptomic Analysis
Source: Parkinsons Dis. 2026 Sep 30;2026:2128634. doi: 10.1155/padi/2128634 (PMC13625826; doi:10.1155/padi/2128634)
Supplement: Supplementary file 1 [file PADI-2026-2128634-s001.zip › Code SI.docx]

#R script for batch correction and normalization pipeline.

############################################################

\# Batch effect correction for Agilent microarray data

\# Method: ComBat (parametric empirical Bayes)

\# Platform: Agilent microarray

\# Input: pre-normalized and log2-transformed expression matrices

\#	(one file per dataset, filename ending in normalize.txt)

############################################################

library(limma)

library(sva)

setwd("PATH_TO_INPUT_FILES")

\# ----------------------------------------------------------

\# Identify normalized input files

\# ----------------------------------------------------------

files <- dir(pattern = "normalize\\.txt$")

\# Exclude merged files

files <- setdiff(files, c("merge.preNorm.txt", "merge.normalize.txt"))

\# ----------------------------------------------------------

\# Extract common genes across datasets

\# ----------------------------------------------------------

geneList <- list()

for (file in files) {

rt <- read.table(file, header = TRUE, sep = "\t", check.names = FALSE)

geneNames <- as.character(rt[, 1])

header <- unlist(strsplit(file, "[.-]"))[1]

geneList[[header]] <- unique(geneNames)

}

interGenes <- Reduce(intersect, geneList)

\# ----------------------------------------------------------

\# Merge expression matrices

\# ----------------------------------------------------------

allTab <- NULL

batchType <- c()

for (i in seq_along(files)) {

rt <- read.table(files[i], header = TRUE, sep = "\t", check.names = FALSE)

rt <- as.matrix(rt)

rownames(rt) <- rt[, 1]

exp <- rt[, 2:ncol(rt)]

data <- matrix(

as.numeric(exp),

nrow = nrow(exp),

dimnames = list(rownames(exp), colnames(exp))

)

\# Collapse multiple probes per gene

data <- avereps(data)

header <- unlist(strsplit(files[i], "[.-]"))[1]

colnames(data) <- paste0(header, "_", colnames(data))

data <- data[interGenes, , drop = FALSE]

if (is.null(allTab)) {

allTab <- data

} else {

allTab <- cbind(allTab, data)

}

batchType <- c(batchType, rep(i, ncol(data)))

}

\# Save pre-correction matrix

write.table(

rbind(geneNames = colnames(allTab), allTab),

file = "merge.preNorm.txt",

sep = "\t",

quote = FALSE,

col.names = FALSE

)

\# ----------------------------------------------------------

\# Apply ComBat batch correction

\# ----------------------------------------------------------

combatOut <- ComBat(

dat = allTab,

batch = batchType,

par.prior = TRUE

)

\# Save corrected expression matrix

write.table(

rbind(geneNames = colnames(combatOut), combatOut),

file = "merge.normalize.txt",

sep = "\t",

quote = FALSE,

col.names = FALSE

)
