# Supplementary material for: Peripheral HMGB1 Downregulation Is Associated With the S100–IL‐17 Axis in Parkinson’s Disease: An Integrated Bulk and Single‐Cell Transcriptomic Analysis
Source: Parkinsons Dis. 2026 Sep 30;2026:2128634. doi: 10.1155/padi/2128634 (PMC13625826; doi:10.1155/padi/2128634)
Supplement: Supplementary file 1 [file PADI-2026-2128634-s001.zip › Supplementary.docx]

**Figure S1. Quality control and variance analysis of single-cell RNA sequencing data. (A) Number of genes, transcripts, and mitochondrial gene percentage for each sample. (B) Principal component analysis (PCA) plot (PC1-PC4) . (C) Association of highly variable genes(HVG) with principal components (PC1–PC4). (D) Permutation test for PCA significance. (E) Selection of HVG. (F) Secondary permutation test for PCA. (G) Scree plot of principal component standard deviations.**

**Figure S2.** T-distributed stochastic neighbor embedding (t-SNE) dimensionality reduction and cell-type annotation for single-cell RNA sequencing data.
