# Supplementary material for: Peripheral HMGB1 Downregulation Is Associated With the S100–IL‐17 Axis in Parkinson’s Disease: An Integrated Bulk and Single‐Cell Transcriptomic Analysis
Source: Parkinsons Dis. 2026 Sep 30;2026:2128634. doi: 10.1155/padi/2128634 (PMC13625826; doi:10.1155/padi/2128634)
Supplement: Supplementary file 1 [file PADI-2026-2128634-s001.zip › Code S2.docx]

#R script for differential expression analysis pipeline.

library(limma)

library(pheatmap)

library(reshape2)

library(ggpubr)

expFile="merge.normalize.txt"	# Expression matrix file

geneFile="gene.txt"	# Target gene list file

diseaseName="Parkinson's Disease" # Disease name for figure labels

setwd("PATH_TO_YOUR_INPUT_FILES") # Set working directory

\# Read expression matrix file

rt=read.table(expFile, header=T, sep="\t", check.names=F)

rt=as.matrix(rt)

rownames(rt)=rt[,1]

exp=rt[,2:ncol(rt)]

dimnames=list(rownames(exp),colnames(exp))

data=matrix(as.numeric(as.matrix(exp)),nrow=nrow(exp),dimnames=dimnames)

data=avereps(data)

\# Read target gene list and subset to those genes

geneRT=read.table(geneFile, header=F, sep="\t", check.names=F)

sameGene=intersect(row.names(data), as.vector(geneRT[,1]))

data=data[sameGene,]

\# Parse sample grouping from column names (format: prefix_dataset_group)

Type=gsub("(.*)\\_(.*)\\_(.*)", "\\3", colnames(data))

data=data[,order(Type)]

Type=gsub("(.*)\\_(.*)\\_(.*)", "\\3", colnames(data)]

Type=ifelse(Type=="Control", "Control", diseaseName)

Type=factor(Type, levels=c("Control", diseaseName))

conNum=sum(Type=="Control")

treatNum=length(Type[Type=="Treat"])

\# Wilcoxon rank-sum test for each gene

sigGeneVec=c()

diffTab=data.frame()

for(i in row.names(data)){

test=wilcox.test(data[i,] ~ Type)

pvalue=test$p.value

conMean=mean(data[i,1:conNum])

treatMean=mean(data[i,(conNum+1):ncol(data)])

diffTab=rbind(diffTab, cbind(Gene=i,conMean,treatMean,pvalue))

}

\# Benjamini-Hochberg FDR correction

pvalue=diffTab[,"pvalue"]

fdr=p.adjust(as.numeric(as.vector(pvalue)), method="fdr")

outTab=cbind(diffTab, fdr=fdr)

\# Filter significant genes (FDR < 0.05)

diffTab=outTab[(as.numeric(as.vector(outTab$fdr))<0.05),]

sigGeneVec=as.vector(diffTab[,"Gene"])

\# Save differential expression results

write.table(diffTab, file="diff.txt", sep="\t", quote=F, row.names=F)

\# Subset expression matrix to significant genes

data=data[sigGeneVec,]

exp=data

\# Heatmap of significant genes

names(Type)=colnames(data)

Type=as.data.frame(Type)

pdf(file="heatmap.pdf", width=7, height=4.5)

pheatmap(data,

annotation=Type,

color = colorRampPalette(c(rep("blue",3), "white", rep("red",3)))(100),

cluster_cols =F,

cluster_rows =T,

scale="row",

show_colnames=F,

show_rownames=T,

fontsize=6,

fontsize_row=7,

fontsize_col=6)

dev.off()

\# Melt expression data for ggplot2

exp=as.data.frame(t(exp))

exp=cbind(exp, Type=Type)

data=melt(exp, id.vars=c("Type"))

colnames(data)=c("Type", "Gene", "Expression")

\# Boxplot with significance annotations

p=ggboxplot(data, x="Gene", y="Expression", fill = "Type",

xlab="",

ylab="Gene expression",

legend.title="Type",

palette = c("#6699FFFF","#E6550DFF"),

outlier.shape = NA,

width=0.8)

p=p+rotate_x_text(60)

p1=p+stat_compare_means(aes(group=Type),

method="wilcox.test",

symnum.args=list(cutpoints = c(0, 0.001, 0.01, 0.05, 1), symbols = c("***", "**", "*", " ")),

label = "p.signif")

pdf(file="boxplot.pdf", width=7, height=5)

print(p1)

dev.off()
